# Cross-sectional study examining the prevalence, correlates and sequencing of electronic cigarette and tobacco use among 11–16-year olds in schools in Wales

**DOI:** 10.1136/bmjopen-2016-012784

**Published:** 2017-02-03

**Authors:** Elen de Lacy, Adam Fletcher, Gillian Hewitt, Simon Murphy, Graham Moore

**Affiliations:** 1 Centre for the Development and Evaluation of Complex Interventions for Public Health Improvement (DECIPHer), School of Social Sciences, Cardiff University, Cardiff, UK; 2 Y Lab, School of Social Sciences, Cardiff University, Cardiff, UK

**Keywords:** Electronic Cigarettes, E-cigarettes, Smoking, Children, Youth

## Abstract

**Objectives:**

To examine the prevalence and frequency of electronic (e)-cigarette use among young people in Wales, associations with socio-demographic characteristics, smoking and other substances and the sequencing of e-cigarette and tobacco use.

**Design:**

A cross-sectional survey of school students in Wales undertaken in 2015.

**Setting:**

87 secondary schools in Wales.

**Participants:**

Students aged 11–16 (n=32 479).

**Results:**

Overall, students were nearly twice as likely to report ever using e-cigarettes (18.5%) as smoking tobacco (10.5%). Use of e-cigarettes at least weekly was 2.7% in the whole sample, rising to 5.7% among those aged 15–16. Almost half (41.8%) of daily smokers reported being regular e-cigarette users. Regular e-cigarette use was more prevalent among current cannabis users (relative risk ratio (RRR)=41.82; 95% CI 33.48 to 52.25)), binge drinkers (RRR=47.88; 95% CI 35.77 to 64.11), users of mephedrone (RRR=32.38; 95% CI 23.05 to 45.52) and laughing gas users (RRR=3.71; 95% CI 3.04 to 4.51). Multivariate analysis combining demographics and smoking status showed that only gender (being male) and tobacco use independently predicted regular use of e-cigarettes (p<0.001). Among weekly smokers who had tried tobacco and e-cigarettes (n=877), the vast majority reported that they tried tobacco before using an e-cigarette (n=727; 82.9%).

**Conclusions:**

Since 2013, youth experimentation with e-cigarettes has grown rapidly in Wales and is now almost twice as common as experimentation with tobacco. Regular use has almost doubled, and is increasing among never and non-smokers. These data suggest that e-cigarette use among youth is an emerging public health issue, even though there remains no evidence that it represents a new pathway into smoking. Mixed methods longitudinal research is needed to explore why young people use e-cigarettes, and to develop interventions to prevent further increases in use.

**Strengths and limitations of this study**

- This is the largest study of young people aged 11–16 in the UK, allowing for analysis of e-cigarette use by age.
- The study examines new measures of regular use of e-cigarettes and assesses the sequencing of e-cigarette and tobacco use.
- The analyses are cross sectional, and hence causal relationships cannot be established.
- The measures of self-reported e-cigarette use are not validated; therefore, the extent to which they capture true prevalence is unclear.

## Background

Electronic (e)-cigarettes are handheld battery-operated devices which aim to simulate conventional cigarettes, and provide inhaled doses of vaporised nicotine. Described as a ‘disruptive technology’ (ie, replacing conventional cigarettes),^1^ these products appeared on the UK market in 2006 and consumption has since grown rapidly, although they are not universally regulated or licensed. There are currently an estimated 2.8 million adult users in the UK, with the majority either ‘dual users’, who use cigarettes as well as e-cigarettes, or ex-smokers.^2^ A recent report by Public Health England^3^ endorsed an estimate that e-cigarettes are 95% safer than tobacco,^4^ although their safety has been contested^5^ and there has been criticism of the primary source of this figure due to its sole basis on expert opinion.^6^ While research on their role for smoking cessation is underdeveloped, e-cigarettes are seen as offering significant potential. They have become the most popular quitting aid for adults in England^7^ and they have been shown to successfully help smokers to stop, or reduce their cigarette consumption.^8–12^ The UK Royal Society for Public Health, Royal College of Physicians and Public Health England now cite their potential for harm reduction and advocate their use for smoking cessation.^3^ ^13^ ^14^

Developing an evidence base of efficacy is made challenging by the rapidly changing nature of these products and e-cigarettes continue to divide opinion regarding their potential public health benefits and harms. For example, the WHO has recommended that policymakers regulate them as tobacco products,^15^ although some UK public health experts have argued against this.^16^ The biggest concern among health professionals, however, is the impact of e-cigarettes on young people. While adult use has been almost exclusively among current smokers and ex-smokers, in recent years there has been a rapid growth in the experimental use of e-cigarettes among younger populations, in the UK and internationally, including among non-smoking, school-aged youth and young adults.^17–21^ For example, the US National Youth Tobacco Survey (NYTS) showed that ever use of e-cigarettes rose rapidly among middle-school youth (1.4% in 2011 to 5.3% in 2015) and high-school youth (4.7% in 2011 to 16.0% in 2015).^17^ Some experts argue that initiation with e-cigarettes may lead young people onto tobacco smoking (so called ‘gateway’ effect), creating a new generation dependent on tobacco.^22–24^ However, while e-cigarettes do not contain tobacco, studies show that exposure to nicotine during childhood and adolescence can inhibit brain development with implications for future emotional and cognitive functions.^25^ ^26^ Hence, if widespread use of e-cigarettes were to occur, it has the potential to become a substantial public health problem in itself.

Despite the rapid growth in youth experimentation, levels of regular use observed to date have been low, with use largely concentrated among existing smokers. For example, in Wales in 2013, experimentation with e-cigarettes had reached the same level as experimentation with tobacco (12.3% and 12.1%) among 11–16-year olds.^27^ However, regular use (defined in that survey as use at least once a month) of e-cigarettes was limited to 1.5% of young people and among never smokers, was very low (0.3%).^27^ Two other youth surveys undertaken in 2013 and 2014 in Scotland and England showed similar levels of experimentation, with low levels of regular use (use at least once a month) of around 1%.^28^ ^29^ The same study in Wales also found that regular e-cigarette use was more likely among those who had ever smoked cannabis, although found no differences according to socio-demographic characteristics.^27^ However, these studies do not consistently examine more frequent measures of use (weekly and daily use) which are stronger measures of ‘established use’.

A number of other studies have found that e-cigarette experimentation is higher among male students^18^ ^30^ and is associated with a number of risk behaviours (tobacco, alcohol, cannabis and shisha use).^19^ ^27^ ^31^ ^32^ For example, a cross-sectional survey in the North West of England found that young people aged 14–17 accessing e-cigarettes were also more likely to use alcohol.^33^ While experimentation with e-cigarettes is increasing, tobacco use continues to fall. In England, by 2014, experimentation with e-cigarettes had grown to 22% overtaking experimentation with conventional cigarettes at 18%.^28^

E-cigarettes are now being promoted in a way which is youth-focused and reminiscent of very successful tobacco advertising.^34–36^ These products are positioned as socially attractive, with celebrity endorsement, use of stylish design and availability in a wide range of flavours.^34^ ^37^ ^38^ While a recent UK study found young people's exposure to e-cigarette advertisements did not increase the appeal of using e-cigarettes or tobacco,^39^ there is an association between the visibility of e-cigarettes in shops and intention to use e-cigarettes.^40^ Hence, there is a concern that the visibility of e-cigarettes in places where the marketing of tobacco has been banned may renormalise smoking, and undermine tobacco control strategies.^41^ Others argue that e-cigarettes may play an important role in ‘de-normalising’ smoking by replacing it as a social alternative.^3^

Public health experts agree that efforts should be made to prevent young people from using e-cigarettes, and there has been a relative consensus on policy interventions targeting this group, such as banning the sale of e-cigarettes to under 18s in all UK countries. However, there are still significant concerns that use of e-cigarettes may limit further declines in tobacco smoking, or even precipitate new increases in youth smoking among a new generation dependent on nicotine via e-cigarette use.^15^ ^24^ ^42^ Harm reduction arguments do not hold where e-cigarettes are used by young people who would otherwise have not taken up smoking. For example, further analysis of the 2011 and 2012 NYTS found that young e-cigarette experimenters had lower odds of abstinence from conventional cigarettes which led to conclusions that e-cigarettes did not support cessation and may encourage conventional cigarette use among US adolescents.^18^ However, this interpretation has been disputed by other researchers due to conclusions which are not justified by the study's results.^43^ ^44^

Concern regarding young people's use of e-cigarettes has focused primarily on whether e-cigarettes increase the likelihood that young people will take up smoking. Previous cross-sectional studies have found an association between e-cigarette use with ‘intention to smoke’, although none have been able to unpack what came first and there are very few published longitudinal studies. In a meta-analysis exploring associations between e-cigarette use and smoking intentions among adolescents and young adults, never smokers who use e-cigarettes are more likely to intend to smoke conventional cigarettes in the future.^45^ Some argue that rather than acting as a ‘gateway’, e-cigarettes may be diverting some young people who would otherwise have become smokers away from tobacco use. To date, one small study following 16 young people in the USA who reported that they had ever used an e-cigarette, but reported no intention to smoke, found that within 1 year, five people had gone on to try at least one puff of a cigarette.^23^ ^46^ However, while the authors acknowledge the limitations of the study, it appeared alongside an editorial which presents it as definitive evidence of a ‘gateway effect’.^23^

While there are a growing number of published studies in the UK and internationally on the prevalence of e-cigarette use among young people, they do not routinely examine frequency of use including weekly and daily e-cigarette use. This study will replicate previous analyses of the 2013 Wales Health Behaviour in School-aged Children (HBSC) survey data on youth e-cigarette use,^27^ and draw on a wider range of measures of e-cigarette use (weekly and daily use), using a more recent cross-sectional survey of secondary school students aged 11–16 undertaken in Wales in 2015. This study therefore provides a more nuanced and up-to-date estimate of the prevalence and frequency of e-cigarette use, including among smokers, ex-smokers and non-smokers, and examines associations with a wide range of other types of substance use (cannabis, alcohol, mephedrone and laughing gas). To address the lack of evidence regarding the sequence of initiating e-cigarette and tobacco use, this study also examines whether those young people who have tried e-cigarettes and tobacco, particularly those who have gone on to become regular smokers, try tobacco or e-cigarettes first to examine pathways into regular use among this age group. These data and analyses will provide more robust evidence to help inform subsequent research and appropriate policy responses in relation to e-cigarettes.

The research objectives are:

- to estimate the prevalence and frequency of e-cigarette use, including among smokers, ex-smokers, never smokers and non-smokers;
- to examine associations of e-cigarette use with socio-demographic characteristics and with use of other substances (tobacco, cannabis, alcohol, mephedrone and laughing gas);
- to examine pathways into regular use among 11–16-year olds by assessing what was tried first (tobacco or e-cigarettes) in those who have tried both.

## Methods

This study uses data collected from the School Health Research Network student health and well-being survey of 87 secondary schools in Wales in 2015. The Wales School Health Research Network (henceforth ‘the Network’) is a multiagency partnership led by the Centre for the Development and Evaluation of Complex Interventions for Public Health Improvement (DECIPHer) at Cardiff University; with Welsh Government, Public Health Wales, Cancer Research UK and 113 secondary schools (as of December 2015), which aims to improve the quality of school-based health improvement research in Wales.

### Study design and recruitment

The survey was a cross-sectional study of students aged 11–16 in network schools which took place between September and December 2015. The survey monitors health behaviours among school-aged students and includes questions from the 2013/2014 Welsh HBSC with additional questions reflecting current policy, practice and research priorities in Wales.

All network schools were invited to participate (n=113). At the time of the survey, network schools represented just over half (53%) of all secondary schools in Wales, with representation in all local authority areas. Schools have joined the Network in three ways. First, those participating in the Welsh HBSC survey in 2013/2014^27^ were invited to join the Network and 60 out of 82 did so. Second, nine schools in South Wales recruited to an HBSC substudy to pilot data linkage methods joined the Network and third, 44 further schools joined in 2015 during a period of open recruitment.

A total of 87 member schools (77%) took part in the survey. Each member school had a designated member of staff who acted as a contact person and they were briefed about the survey via emails, newsletters and at an event for schools in June 2015. The survey was an online, closed-response, self-complete survey, available in English and Welsh and schools managed its implementation using their own IT facilities. Schools were asked to include all students, but if this was not possible, to include a minimum of two randomly selected, mixed ability classes per year. Schools were advised to oversee students taking the survey, but that staff should remain at the front of the room unless a student asked for help.

### Measures

#### Socio-demographic characteristics

Students indicated their sex, year and month of birth. To measure socioeconomic status, children completed the Family Affluence Scale (FAS).^35^ The FAS comprises of measures of bedroom occupancy, car and computer ownership and family holidays. Items on dishwashers and bathrooms were introduced in 2013, due to concerns that some items (ie, computer ownership) became less differentiated by socioeconomic status over time. These were summed to give an overall measure of family affluence. Young people were also asked which of the following best described them: white; mixed race; Asian or Asian British; black or black British; Chinese or other.

#### Use of e-cigarettes

Students were asked if they had ever used an e-cigarette, with response options of: ‘I have never tried e-cigarettes’; ‘I have used e-cigarettes once’ or ‘I have tried e-cigarettes more than once’. If young people had tried e-cigarettes more than once, they were then asked ‘How often do you use e-cigarettes at present?’ with response options of: ‘Every day’; ‘At least once a week, but not every day; or ‘less than once a week’. For analyses, responses of; ‘At least once a week, but not every day’ and ‘Every day’ were combined to measure ‘regular use’. ‘Occasional use’ was measured with the response option of ‘less than once a week’. Participants were also asked; ‘At what age did you first do the following things?’ with response options of: ‘Never’; or the option of a range of ages (11–16) if ‘Smoked a cigarette (more than a puff)’ and/or ‘Used an e-cigarette’. These were used to derive percentages of ever use for tobacco and e-cigarettes (ie, never vs all other responses).

#### Frequency of current smoking

Frequency of current smoking was measured by asking young people how often they smoked tobacco at present, with five response options: ‘I do not want to answer’; ‘I do not smoke’; ‘Less than once a week’; ‘At least once a week, but not every day’; ‘Every day’. A binary variable (‘ever smoked’) was created from the question on age of first use of tobacco (ie, never vs all other responses). Young people who did not currently smoke regularly were asked if they ever had smoked weekly or more. Those young people who said yes, but currently stated that they did not smoke, were classed as ex-smokers.

#### Sequencing of e-cigarette or tobacco use

The subsample of young people who reported having tried tobacco and e-cigarettes were asked an additional question which simply asked them to indicate which they had used first (tobacco or e-cigarettes).

#### Alcohol use per drinking session

Alcohol use per drinking session was measured by asking young people ‘How many drinks containing alcohol do you have on a typical day when you are drinking?’ with response options of; ‘I never drink alcohol’; ‘Less than one drink’; ‘1 drink’; ‘2 drinks’; ‘3 drinks’; ‘4 drinks’; ‘5 or more drinks’; ‘I do not want to answer’. These options were combined to produce a three-category alcohol drinks per session variable (none, 1–4 and 5+). ‘Binge drinking’ was defined as having five or more drinks per drinking session.

#### Prevalence and frequency of cannabis use

Students were asked; ‘Have you ever taken cannabis?’ with two subquestions; ‘In your life’ or ‘In the last 30 days’. They were then asked to indicate use on how many days with response options of; ‘Never’; ‘1–2 days’; ‘3–5 days’; ‘6–9 days’; ‘10–19 days’; ‘20–29 days’; ‘30 days or more’; ‘I do not want to answer’. For analyses, a binary variable (‘cannabis ever use’) was produced and a three-category past month cannabis use variable was also examined (never, less than daily and daily) as a marker of current use.

#### Use of novel psychoactive substances: mephedrone and laughing gas

Students were asked ‘In your life have you ever tried mephedrone (also called ‘m-cat’ and ‘meow meow’)?’ with response options of; ‘Yes’; ‘No’; ‘I do not want to answer’. For analyses, a binary variable (‘mephedrone ever use’) was produced. Students were asked ‘In your life have you ever tried inhaling laughing gas (also called nitrous oxide, ‘balloons’ and ‘whippits’)?’ with response options of; ‘Yes’; ‘No’; ‘I do not want to answer’. For analyses, a binary variable (‘laughing gas ever use’) was produced.

### Research ethics and consent

Schools returned a registration form indicating their intention to participate in the study. Schools informed parents about the survey using two of three methods (letters sent home with students or via email, and a text message notification about the letter) and parents had the option of withdrawing their child from data collection (‘opt-out’ consent procedure). The survey was voluntary and completed anonymously. The first question asked students for their consent to participate and if they said no, the survey automatically closed. Schools were provided with information and slides to share with students in advance of the survey.

### Statistical analysis

First, the percentages of young people who have ever used e-cigarettes and who use them at least weekly within each school year are presented graphically, alongside the percentage who report ever use and weekly use of tobacco. Second, the percentages of never smokers who report weekly e-cigarette use are presented graphically by year group alongside the percentage of non-smokers reporting weekly e-cigarette use. A ‘never smoker’ is defined as a young person who has never smoked and a ‘non-smoker’ is defined as a young person who does not currently smoke (but may have previously). These groups are analysed separately to understand whether e-cigarettes are being used by young people who never tried nicotine or have previously tried nicotine. Missing data and responses of ‘I do not want to answer’ are excluded from analyses as >95% of young people provided sufficient data to categorise their smoking status. Binary logistic regression models are used to assess associations of demographic variables, tobacco use and other substance use with ever e-cigarette use. Multinomial logistic regression models are used to examine associations of demographic variables, tobacco use and other substance use with e-cigarette use, with ‘occasional’ and ‘regular’ use compared against the reference category of ‘I don't use e-cigarettes’. Multivariate models then combine demographic characteristics, smoking status and other substance use with e-cigarette use, with ‘ever’, ‘occasional’ and ‘regular’ use compared against the reference category of ‘never used an e-cigarette’. To examine the sequence of initiating e-cigarette and tobacco use, frequency of using e-cigarettes/tobacco first (asked of the subsample of young people who had reported use of tobacco and e-cigarettes) was calculated for the entire subsample and by smoking status. All models are adjusted for clustering at the school-level and the ‘svy’ setting in Stata is used to account for the non-independence of individuals within clusters. Data from Year 11 are analysed separately to enable comparability with other studies.^29^ Analyses were conducted using Stata V.14.

## Results

Of the 32 479 students who began the survey, 30 917 (95.2%) students provided responses to the questions on their smoking status, while 28 634 (88.2%) completed the FAS questionnaire (the last items in the survey). Almost one in five young people (18.5%) aged 11–16 had used an e-cigarette at least once and 2.7% students reported using them at least weekly. Overall, 10.5% (n=3180) of young people reported ever having smoked, while 2.3% reported daily smoking. Among Year 11 students (aged 15–16), the prevalence of ever using e-cigarettes was 37.3% (n=1858), with 5.7% (n=187) reporting they used them regularly (weekly or more). Ever smoking was reported by 26.5% (n=1324) and daily smoking by 6.3% (n=320) of Year 11 students (table 1).

**Table 1:** Socio-demographic and behavioural characteristics of participants

School Health Research Network survey measures (no. of respondents for ages 11–16/15–16)	Years 7–11 (aged 11–16)	Year 11 (aged 15–16)
Mean (SD) age (n=32 386)/(n=5353)	13.6 (1.4)	15.7 (0.3)
Female % (n) (n=31 334)/(n=5373)	52.0 (1881)	53.4 (2871)
BME % (n) (n=31 614)/(n=5290)	11.1 (3521)	12.2 (645)
Mean (SD) FAS (n=28 661)/(n=4674)	14.9 (2.0)	14.9 (2.0)
Ever smoked % (n) (n=30 327)/(n=5001)	10.5 (3182)	26.5 (1325)
Smoking status % (n) (n=30 353)/(n=5050)		
Don't	94.0 (29 045)	84.6 (4276)
<Weekly	1.5 (458)	3.9 (197)
Weekly	1.0 (331)	2.5 (124)
Daily	2.3 (717)	6.3 (320)
Ex-smoker	1.2 (366)	2.7 (135)
Ever used an e-cigarette % (n) (n=30 259)/(n=4990)	18.5 (5587)	37.3 (1860)
Current e-cigarette use % (n) (n=30 917)/(n=5060)		
Don't	95.6 (29 434)	91.0 (4602)
<Weekly	1.8 (542)	3.3 (169)
Weekly	1.3 (399)	2.4 (121)
Daily	1.4 (423)	3.3 (166)
Cannabis ever use % (n) (n=26 858)/(n=4407)	**5**.0 (1332)	14.8 (653)
Cannabis use in past month % (n) (n=26 902)/(n=4426)		
Never	97.1 (26 141)	92.1 (4078)
Less than daily	2.0 (534)	5.6 (249)
Daily	0.8 (227)	2.2 (99)
Mephedrone ever use % (n) (n=26 763)/(n=4447)	1.3 (342)	2.6 (117)
Laughing gas ever use % (n) (n=26 772)/(n=4445)	27.1 (7258)	27.4 (1219)
Alcoholic drinks per session % (n) (n=30 174)/(n=4862)		
None	72.0 (21 772)	40.1 (1949)
1–4	21.6 (6520)	39.1 (1902)
5+	6.2 (1882)	20.8 (1011)

BME, black and minority ethnicity; FAS, Family Affluence Scale.

**Figure 1:**
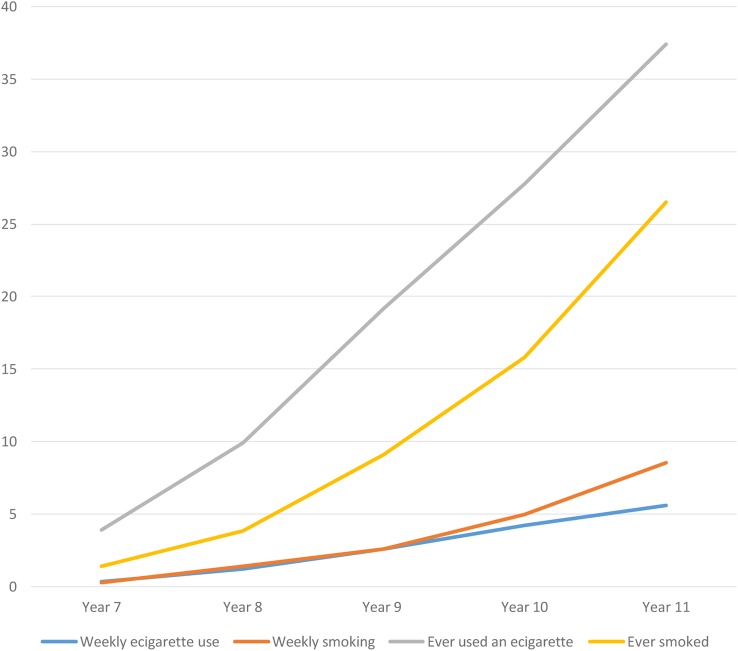
Percentage of young people reporting ever and weekly use of tobacco or e-cigarettes by school-year group.

Figure 1 shows the proportion of young people who report ever and weekly use of e-cigarettes or tobacco by school-year group. All these rates rise in parallel, with weekly smoking and e-cigarette use very similar among younger age groups until school-year 11 (aged 15–16), when it is overtaken by smoking.

**Figure 2:**
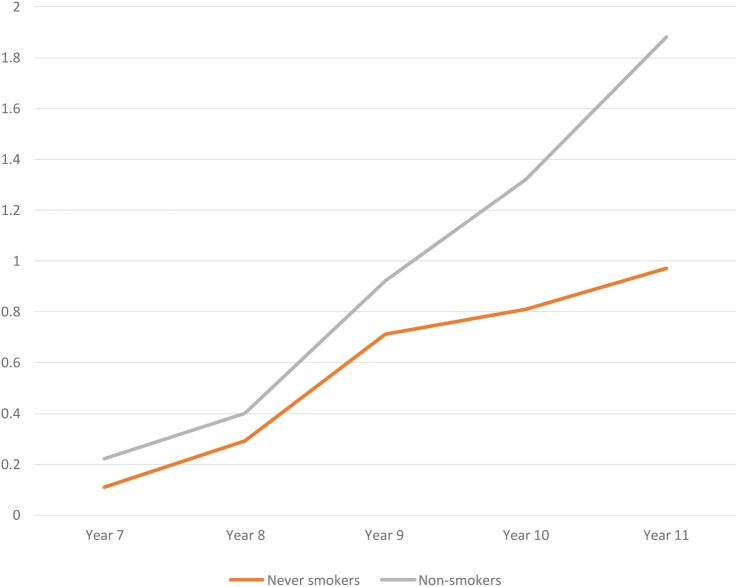
Percentage of never smokers and non-smokers reporting weekly e-cigarette use by school-year group.

**Figure 3:**
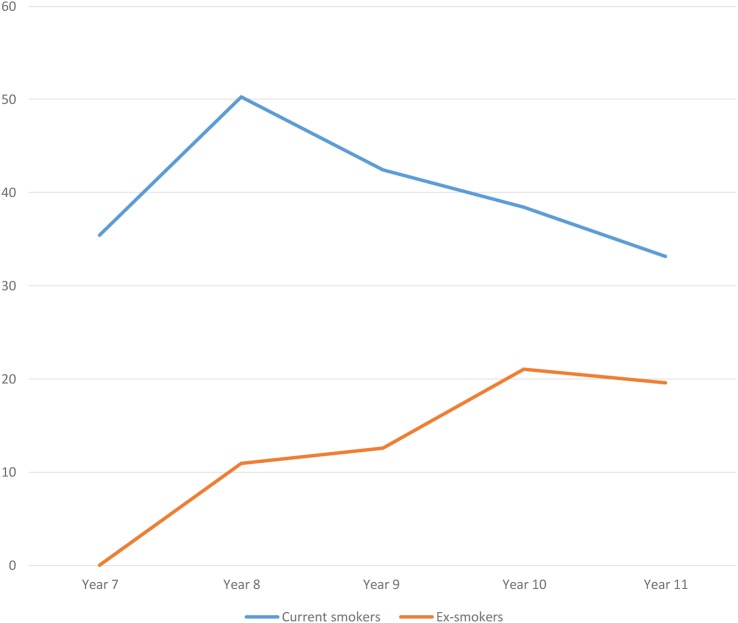
Percentage of smokers and ex-smokers reporting weekly e-cigarette use by school-year group.

Figure 2 shows weekly e-cigarette use among never smokers and non-smokers by year group. While regular use among never smokers and non-smokers was low, one in 100 reported regular use. This figure rose to 2% where it extended to include occasional (less than weekly) users of e-cigarettes. In figure 2, the proportion of non-smokers who regularly used e-cigarettes increased with age, while in figure 3, the percentage of smokers using e-cigarettes did not.

In the sample, overall (11–16 years) and among 15–16-year olds, young male students were significantly more likely than young female students to have used an e-cigarette, while young people from minority ethnic backgrounds were also more likely to report use. There was very clear patterning according to smoking characteristics with the vast majority of ever smokers and current smokers, as well as ever and current cannabis users, reporting that they had also tried an e-cigarette. Alcohol, mephedrone and laughing gas use was also strongly associated with ever e-cigarette use (table 2).

**Table 2:** Univariate associations of socio-demographic characteristics and use of other substances with ever use of e-cigarettes

	All year groups (11–16 years)		Year 11 (15–16 years) only	
	% (n)	RRR (95% CI)	% (n)	RRR (95% CI)
Sex				
Male	19.3 (2795)	**0.88 (0.82 to 0.96)**	37.6 (866)	**0.96 (0.82 to 1.12)**
Female	17.7 (2792)	1.00	37.2 (994)	1.00
Ethnicity				
White	18.6 (4896)	1.00	37.6 (1635)	1.00
BME	19.1 (602)	1.03 (0.82 to 1.31)	35.4 (203)	**0.91 (0.61 to 1.35)**
FAS	–	**0.96 (0.94 to 0.98)**	–	**0.95 (0.93 to 0.98)**
Ever smoked				
No	10.4 (2789)	1.00	19.9 (722)	1.00
Yes	85.9 (2712)	**52.96 (44.31 to 63.29)**	85.0 (1122)	**23.08 (17.48 to 30.48)**
Smoking status				
I do not smoke	13.6 (3835)	1.00	27.7 (1149)	1.00
Less than once a week	84.6 (366)	**34.80 (24.87 to 48.70)**	82.1 (156)	**12.09 (7.23 to 20.23)**
At least once a week (but not every day)	90.8 (286)	**65.07 (39.03 to 108.47)**	89.0 (109)	**22.10 (11.85 to 41.19)**
Every day	92.4 (607)	**74.36 (53.76 to 102.86)**	92.6 (280)	**32.08 (19.22 to 53.55)**
Ex-smoker	85.4 (298)	**37.97 (27.34 to 52.74)**	85.0 (111)	**14.62 (8.98 to 23.82)**
Ever used cannabis				
No	14.1 (3496)	1.00	27.3 (998)	1.00
Yes	86.8 (1121)	**39.60 (30.92 to 50.71)**	87.2 (561)	**18.24 (13.56 to 24.55)**
Cannabis use				
None	15.7 (3995)	1.00	31.7 (1263)	1.00
Less than daily	90.7 (467)	**50.47 (34.71 to 73.38)**	92.3 (225)	**25.78 (14.87 to 44.71)**
Every day	86.7 (184)	**33.14 (21.92 to 50.10)**	89.7 (85)	**18.51 (9.89 to 34.62)**
Ever used mephedrone				
No	17.3 (4450)	1.00	35.8 (1511)	1.00
Yes	73.8 (233)	**13.02 (9.20 to 18.42)**	83.0 (93)	**8.89 (5.48 to 14.43)**
Ever used laughing gas				
No	13.0 (2479)	1.00	30.8 (972)	1.00
Yes	31.9 (2255)	**3.18 (2.92 to 3.46)**	53.2 (634)	**2.58 (2.21 to 3.01)**
Alcohol				
None	7.2 (1551)	1.00	14.4 (279)	1.00
1–5	37.2 (2349)	**7.63 (6.93 to 8.41)**	40.5 (764)	**4.04 (3.44 to 4.75)**
5+	72.0 (1326)	**33.51 (28.96 to 38.77)**	71.5 (713)	**15.08 (11.96 to 19.03)**

Bold values signify significant findings p<0.05.

BME, black and minority ethnicity; FAS, Family Affluence Scale; RRR, relative risk ratio.

As indicated in table 3, regular use of e-cigarettes was also more common in young male students, minority ethnic groups and young people from poor families. Almost a third of ever smokers reported at least occasional use of e-cigarettes, compared with 1.2% of never smokers. Almost half of daily smokers reported regular use of e-cigarettes, compared with 2% of non-smokers. E-cigarette use was reported by about one in four ex-smokers. Cannabis use was strongly associated with current e-cigarette use, along with binge drinking, mephedrone use and laughing gas use.

**Table 3:** Univariate associations of socio-demographic characteristics and use of other substances with frequency of e-cigarette use

	Occasional use (less than weekly)		Regular use (weekly or more)	
	n (%)	RRR (95% CI)	n (%)	RRR (95% CI)
Gender				
Male	284 (1.9)	**0.81 (0.67 to 0.99)**	477 (3.1)	**0.65 (0.52 to 0.81)**
Female	258 (1.6)	1.00	345 (2.1)	1.00
Ethnicity				
White	467 (1.7)	1.00	665 (2.5)	1.00
BME	65 (1.8)	1.18 (0.86 to 1.61)	135 (4.2)	**1.71 (1.28 to 2.29)**
FAS	–	0.99 (0.95 to 1.04)	–	**0.94 (0.89 to 0.99)**
Ever smoked cigarettes				
No	179 (0.7)	1.00	141 (0.5)	1.00
Yes	343 (11.0)	**23.93 (19.49 to 29.37)**	640 (20.0)	**56.67 (44.40 to 72.33)**
Frequency of current tobacco use				
I do not smoke	300 (1.0)	1.00	248 (0.9)	1.00
Less than once a week	81 (18.1)	**28.39 (21.42 to 37.63)**	95 (21.5)	**40.28 (30.19 to 51.22)**
At least once a week (but not every day)	43 (13.6)	**22.20 (14.81 to 33.26)**	93 (29.3)	**58.07 (39.45 to 85.48)**
Every day	67 (10.0)	**19.61 (13.92 to 27.63)**	292 (41.8)	**103.37 (77.94 to 137.10)**
Ex-smoker	25 (7.1)	**8.70 (5.52 to 13.70)**	61 (17.5)	**25.67 (18.16 to 36.31)**
Ever cannabis use				
No	292 (1.1)	1.00	267 (1.1)	1.00
Yes	146 (11.4)	**15.34 (11.87 to 19.83)**	364 (26.8)	**41.82 (33.48 to 52.25)**
Cannabis use				
None	351 (1.3)	1.00	355 (1.4)	1.00
Less than daily	75 (14.4)	**19.25 (14.79 to 25.05)**	173 (32.7)	**43.90 (32.53 to 59.23)**
Every day	16 (7.5)	**11.61 (5.80 to 23.27)**	106 (47.0)	**76.07 (49.91 to 115.92)**
Ever used mephedrone				
No	426 (1.6)	1.00	531 (2.0)	1.00
Yes	24 (7.8)	**7.69 (5.05 to 11.71)**	126 (36.5)	**32.39 (23.05 to 45.52)**
Ever used laughing gas				
No	208 (1.1)	1.00	294 (1.5)	1.00
Yes	253 (3.5)	**3.53 (2.97 to 4.18)**	376 (5.0)	**3.71 (3.04 to 4.51)**
Alcoholic drinks per session				
None	110 (0.5)	1.00	114 (0.5)	1.00
1–5	223 (3.4)	**7.38 (5.76 to 9.46)**	304 (4.7)	**9.71 (7.24 to 13.03)**
5+	172 (9.3)	**24.96 (19.07 to 32.65)**	342 (17.6)	**47.88 (35.77 to 64.11)**

Bold values signify significant findings p<0.05.

BME, black and minority ethnicity; FAS, Family Affluence Scale; RRR; relative risk ratio.

Among young people aged 15–16, e-cigarettes were regularly used by the majority of frequent smokers (59.0%), and current use of e-cigarettes was 3.9% among non-smokers. While the percentage of non-smokers using e-cigarettes was greater in older youth than among the population as a whole, the percentage of smokers using e-cigarettes was slightly less. ORs also showed a strong association between cannabis use, binge drinking, mephedrone use and laughing gas use at age 15–16 (table 4).

**Table 4:** Associations between frequency of e-cigarette use and socio-demographic and smoking characteristics of participants in Year 11 (15–16 years)

	Occasional use (less than weekly)		Regular use (weekly or more)	
	n (%)	RRR (95% CI)	n (%)	RRR (95% CI)
Gender				
Male	85 (3.7)	1.00	182 (7.7)	1.00
Female	84 (3.1)	**0.80 (0.56 to 1.15)**	106 (3.8)	**0.47 (0.24 to 0.65)**
Ethnicity				
White	150 (3.4)	1.00	237 (5.4)	1.00
BME	16 (2.8)	0.82 (0.43 to 1.58)	42 (7.2)	**1.36 (0.89 to 2.09)**
FAS	–	0.99 (0.93 to 1.05)	–	**0.89 (0.85 to 0.96)**
Ever smoked cigarettes				
No	44 (1.2)	1.00	35 (1.0)	1.00
Yes	122 (9.5)	**10.51 (7.03 to 15.70)**	240 (18.0)	**25.98 (16.99 to 39.72)**
Frequency of current tobacco use				
I do not smoke	83 (2.0)	1.00	80 (1.9)	1.00
Less than once a week	32 (16.6)	**12.17 (7.82 to 18.94)**	32 (16.5)	**12.62 (7.95 to 20.06)**
At least once a week (but not every day)	13 (11.1)	**7.68 (3.83 to 15.41)**	25 (21.0)	**15.33 (8.95 to 26.26)**
Every day	28 (9.4)	**8.53 (5.06 to 14.38)**	119 (38.0)	**37.62 (24.79 to 57.10)**
Ex-smoker	9 (7.1)	**4.55 (2.02 to 10.26)**	25 (19.6)	**13.11 (8.29 to 20.76)**
Ever cannabis use				
No	87 (2.4)	1.00	63 (10.0)	1.00
Yes	73 (1.9)	**6.00 (4.11 to 8.76)**	152 (23.1)	**17.25 (12.68 to 23.46)**
Cannabis use				
None	113 (2.8)	1.00	115 (2.8)	1.00
Less than daily	30 (12.2)	**6.77 (4.84 to 9.46)**	67 (27.2)	**14.85 (10.03 to 21.99)**
Every day	9 (10.1)	**7.43 (3.22 to 17.15)**	45 (47.6)	**36.49 (20.24 to 65.78)**
Ever used mephedrone				
No	142 (3.4)	1.00	188 (4.4)	1.00
Yes	10 (9.8)	**4.82 (2.40 to 9.71)**	46 (39.2)	**16.76 (9.38 to 29.94)**
Ever used laughing gas				
No	83 (2.6)	1.00	71 (6.0)	1.00
Yes	118 (3.7)	**2.54 (1.84 to 3.51)**	119 (9.5)	**2.99 (2.20 to 4.07)**
Alcoholic drinks per session				
None	20 (1.0)	1.00	26 (1.3)	1.00
1–5	62 (3.3)	**3.36 (1.94 to 5.82)**	90 (4.6)	**3.75 (2.23 to 6.31)**
5+	82 (8.3)	**10.13 (6.20 to 16.54)**	152 (14.9)	**14.44 (8.49 to 24.57)**

Bold values signify significant findings p<0.05.

BME, black and minority ethnicity; FAS, Family Affluence Scale, RRR; relative risk ratio.

In a multivariate model combining demographics and smoking status, only gender (being male) (p<0.001) and tobacco use (p<0.001) were associated with ever use of e-cigarettes, with no significant differences according to family affluence or ethnicity due to smoking being more common among those groups. Gender (p<0.001) and tobacco use (p<0.001) were also independently associated with occasional and regular use of e-cigarettes (table 5).

**Table 5:** Multivariate associations between e-cigarette use and socio-demographic and smoking status among all participants (aged 11–16 years)

	Ever use	Frequency of use	
		Occasional use (less than weekly)	Regular use (weekly or more)
	OR (95% CI)	RRR (95% CI)	RRR (95% CI)
Gender			
Male	**0.83 (0.75 to 0.91)**	**0.71 (0.57 to 0.87)**	**0.50 (0.38 to 0.66)**
Female	–	–	–
Ethnicity			
White	–	–	–
BME	0.91 (0.75 to 1.11)	1.04 (0.74 to 1.46)	1.26 (0.97 to 1.65)
FAS	0.99 (0.97 to 1.01)	1.03 (0.98 to 1.07)	1.02 (0.97 to 1.07)
Frequency of current tobacco use			
I do not smoke	–	–	–
Less than once a week	**36.66 (26.58 to 50.57)**	**31.93 (23.73 to 42.95)**	**31.93 (23.73 to 42.95)**
At least once a week (but not every day)	**75.53 (47.70 to 119.61)**	**23.04 (14.64 to 36.26)**	**72.38 (46.87 to 111.80)**
Every day	**70.29 (50.71 to 97.44)**	**22.51 (15.56 to 32.55)**	**107.17 (76.73 to 149.70)**
Ex-smoker	**37.87 (26.77 to 53.57)**	**9.05 (5.61 to 14.59)**	**28.07 (18.88 to 41.74)**

Bold values signify significant findings p<0.05.

BME, black and minority ethnicity; FAS, Family Affluence Scale; RRR, relative risk ratio.

Among young people aged 15–16, only smoking status (p<0.001) independently predicted ever and occasional use of e-cigarettes in a multivariate model combining demographics and smoking status. Regular use was also independently predicted by gender (p<0.001) with no differences according to ethnicity or family affluence (table 6).

**Table 6:** Multivariate associations between e-cigarette use and socio-demographic and smoking status of participants aged 15–16 years

	Ever use	Frequency of use	
		Occasional use (less than weekly)	Regular use (weekly or more)
	OR (95% CI)	RRR (95% CI)	RRR (95% CI)
Gender			
Male	0.91 (0.77 to 1.08)	0.64 (0.44 to 0.95)	**0.31 (0.21 to 0.46)**
Female	–	–	–
Ethnicity			
White	–	–	–
BME	0.80 (0.56 to 1.13)	0.79 (0.41 to 1.54)	1.05 (0.70 to 1.56)
FAS	1.00 (0.96 to 1.04)	1.02 (0.96 to 1.08)	1.01 (0.94 to 1.08)
Frequency of current tobacco use			
I do not smoke	–	–	–
Less than once a week	**12.85 (7.66 to 21.56)**	**13.99 (8.73 to 22.42)**	**16.29 (10.07 to 26.36)**
At least once a week (but not every day)	**22.37 (12.26 to 40.81)**	**8.0 (3.60 to 17.75)**	**22.95 (13.10 to 40.17)**
Every day	**30.69 (18.83 to 50.04)**	**10.08 (6.15 to 16.52)**	**43.53 (28.25 to 67.06)**
Ex-smoker	**14.98 (9.11 to 24.65)**	**3.88 (1.48 to 10.14)**	**14.85 (9.24 to 23.86)**

Bold values signify significant findings p<0.05.

BME, black and minority ethnicity; FAS, Family Affluence Scale, RRR; relative risk ratio.

Among all students aged 11–16, a subsample of 2640 students reported that they had tried tobacco and e-cigarettes. Of these young people, most (n=1754; 66.4%) reported that they tried tobacco first. The vast majority of weekly smokers reported that they tried tobacco before using an e-cigarette (n=727; 82.9%). Among occasional (less than weekly smokers), a large majority (n=236, 68.0%) also reported that they tried tobacco first. Among ex-smokers, 78.3% (n=223) reported that they had tried tobacco first. Non-smokers (n=995) who had tried e-cigarettes and tobacco were evenly divided according to which of these they tried first (tobacco first, n=489; 49.2%).

## Discussion

Since 2013, there has been a marked growth in experimentation with and regular use of e-cigarettes among secondary school students in Wales. Experimentation with e-cigarettes is now almost twice as common as experimentation with tobacco (18.5% vs 10.5%), a substantial shift from 2013, when a similar study in Wales found experimentation levels were approximately equal (12.3% and 12.1%).^27^ Similar findings have also been reported among US high school students in 2015, where current use of e-cigarettes was three times higher than tobacco (16% vs 6%).^17^

Although regular e-cigarette use remains relatively low in Wales, it is growing at a faster rate than experimental use. Regular e-cigarette use has almost doubled since 2013, rising from 1.5% (monthly use)^27^ to 2.7% (weekly use) in 2015. It is difficult to accurately compare regular use between the two studies as the HBSC survey in 2013 did not measure weekly e-cigarette use. Hence, the almost doubling of regular use, despite use of a far more stringent measure in 2015, indicates that this is likely to be an underestimate of the true rate of growth. Where including occasional e-cigarette use (less than weekly) in 2015 estimates, while only 1.5% had used an e-cigarette in the past month in 2013 (roughly one in 10 of the 12.3% of ever users),^27^ this rate has increased to 4.4% (roughly one in four of the 18.5% ever users) in 2015, which suggests that the proportion of experimenters going on to use e-cigarettes more frequently is growing. However, there is no evidence that smoking has grown in line with the rapid rise in e-cigarette use, with estimates of ever smoking falling slightly, while estimates of regular smoking remained similar to 2013. There continues to be a need to monitor tobacco and e-cigarette prevalence to examine whether e-cigarettes use impacts, positively or negatively, on smoking trajectories of young people's tobacco use as regular use gains traction.

The rapid growth in youth e-cigarette use in Wales suggests that these products are highly visible and easily available to young people. Although there are restrictions on the sale of e-cigarettes to minors across the UK, they are widely available online and there is little control on the marketing of these products in comparison to tobacco products. From May 2016 the European Union (EU) Tobacco Products Directive (TPD) will prohibit cross-border advertising, require e-cigarettes which are unlicensed to carry health warnings and limit their nicotine strength. These restrictions aim to reduce the visibility of e-cigarettes to young people and address risk perceptions. However, there is limited evidence of young people's risk perceptions of e-cigarettes in the UK, with some studies suggesting they already view e-cigarettes as highly risky.^47^

The major concern for public health professionals and policymakers remains that e-cigarettes could be a new route into nicotine addiction for a large number of young never smokers, if widespread regular use occurs. Evidence from animal models suggests that nicotine use during adolescence can inhibit brain development, with potential implications for later emotional and cognitive functions.^26^ While regular e-cigarette use among never smokers and non-smokers remains low, there has been a substantial relative increase in use. In 2013, only one in 300 never smokers had ever used an e-cigarette in the past month,^27^ whereas in 2015, according to a more stringent measure of regular use (ie, weekly or daily use), one in 100 reported regular use.

Consistent with previous research,^19^ ^27^ ^31^ ^32^ strong links between tobacco and other substances were observed in this study, and it is known that young people who engage in one risk behaviour are more likely to be involved in additional risk behaviours.^48^ E-cigarette use was also more commonly reported by male students which is consistent with previous studies from the USA^18^ and Korea^30^ on youth e-cigarette use. There has also been a substantial increase in the proportion of non-smokers who regularly use e-cigarettes, despite the more stringent measure of regular use in this paper (ie, weekly use) by comparison to our earlier analysis (ie, any use in the past month). Therefore, if this rapid growth in regular use is left unmonitored, young people's use of e-cigarettes is on course to become a public health problem regardless of its links to smoking, and hence, it is important to understand how this upward trajectory might be prevented.

There has been a twofold increase in ‘dual use’ of e-cigarettes since 2013, with almost half of daily young smokers now reporting being regular e-cigarette users. This could indicate that some smokers are using e-cigarettes as a means of quitting smoking or reducing their harm, and almost one in five ex-smokers reported regular use of e-cigarettes in this study. While there is evidence that they have helped adult smokers to stop, or reduce their cigarette consumption^8–12^ there are no studies which have explored their use for adolescent cessation. However, there is no evidence to suggest that use of e-cigarettes have accelerated downward trajectories in smoking, in fact, it is possible that these young people who would otherwise have stopped smoking altogether are continuing to use nicotine via e-cigarettes. Those young people may simply be using e-cigarettes as a way of covertly obtaining nicotine where policies prohibit the use of conventional cigarettes (eg, in school), although qualitative research is needed to fully understand this use.

The vast majority of young people in this study who had tried cigarettes and e-cigarettes reported that they had tried tobacco first, and this was particularly the case for established smokers. Hence it appears that tobacco use is still the most common pathway into nicotine addiction and regular smoking and provides little support for the hypothesis that e-cigarettes are acting as a pathway into smoking.^27^ ^49^ In fact, tobacco is perhaps more likely to be acting as a route into e-cigarette use, rather than the other way around**.** There are a number of cross-sectional studies which have found an association between e-cigarette use and intentions to smoke substances such as tobacco.^45^ However, longitudinal studies are needed to fully explore this and unpick the temporal relationship between e-cigarette and tobacco use.

This study benefits from a large sample of young people aged 11–16 across Wales, allowing for analysis of e-cigarette use by age. Though not sampled with the primary aim of achieving national representativeness, our comparisons of the demographic make-up of the sample suggest that it is directly comparable to earlier nationally representative samples in Wales. However, there a number of limitations which should be considered. In common with all surveys of e-cigarette use, the measures of self-reported e-cigarette are not validated, so the extent to which they capture true prevalence is unclear. There may also have been an overestimate of the number of young people using laughing gas, as some may have interpreted ‘balloons’ as helium balloons as opposed to nitrous oxide. The analyses are cross sectional, and hence causal relationships cannot be established.

## Conclusion

In Wales, young people's use of e-cigarettes has increased rapidly since 2013, including rates of regular e-cigarette use, which have almost doubled in that period. Trying e-cigarettes has become almost twice as common as experimentation with tobacco for school-aged youth in Wales. Despite the strong association between e-cigarette use and smoking status, regular e-cigarette use among never smokers and non-smokers is increasing. This suggests that if left unchecked, young people's e-cigarette use could become a public health problem in itself, regardless of its links to smoking. There is a need to examine youth e-cigarette and tobacco use longitudinally to test how a growth in e-cigarette use may impact, positively or negatively, on smoking uptake, overall and for key subgroups. There is also a need to understand the impacts of current legislative measures like the EU TPD in stemming the increase in young people's e-cigarette use. To develop interventions to prevent further increases in e-cigarette use, qualitative data is also required to understand e-cigarette use from young people's perspective and explore why they start, and continue, to use them.
